# Population genetic structure of *Bemisia tabaci* MED (Hemiptera: Aleyrodidae) in Korea

**DOI:** 10.1371/journal.pone.0220327

**Published:** 2019-07-25

**Authors:** Yujeong Park, Hwa Yeun Nam, Sunghoon Baek, Si Hyeock Lee, Joon-Ho Lee

**Affiliations:** 1 Entomology Program, Department of Agricultural Biotechnology, Seoul National University, Seoul, Republic of Korea; 2 Research Institute of Agriculture and Life Sciences, Seoul National University, Seoul, Republic of Korea; University of Crete, GREECE

## Abstract

The sweet potato whitefly, *Bemisia tabaci* (Gennadius) (Hemiptera: Aleyrodidae) is a major agricultural pest that causes economic damages worldwide. In particular, *B*. *tabaci* MED (Mediterranean) has resulted in serious economic losses in tomato production of Korea. In this study, 1,145 *B*. *tabaci* MED females from 35 tomato greenhouses in different geographic regions were collected from 2016 to 2018 (17 populations in 2016, 13 in 2017, and five in 2018) and analyzed to investigate their population genetic structures using eight microsatellite markers. The average number of alleles per population (*N*_A_) ranged from 2.000 to 5.875, the expected heterozygosity (*H*_E_) ranged from 0.218 to 0.600, the observed heterozygosity (*H*_O_) ranged from 0.061 to 0.580, and the fixation index inbreeding coefficient (*F*_IS_) ranged from -0.391 to 0.872 over the three years of the study. Some significant correlation (*p* < 0.05) was present between genetic differentiations (*F*_ST_) and geographical distance, and a comparatively high proportion of variation was found among the *B*. *tabaci* MED populations. The *B*. *tabaci* MED populations were divided into two well-differentiated genetic clusters within different geographic regions. Interestingly, its genetic structures converged into one genetic cluster during just one year. The reasons for this genetic change were speculated to arise from different fitness, insecticide resistance, and insect movement by human activities.

## Introduction

The sweet potato whitefly, *Bemisia tabaci* (Gennadius) (Hemiptera: Aleyrodidae) is a major agricultural insect pest that is distributed worldwide. *B*. *tabaci* has an extremely broad host range [1] and causes serious damage to diverse host plant species. *B*. *tabaci* is also a vector for more than 100 pathogenic plant viruses [2], particularly known to be a vector for begomoviruses [3], and a major vector for tomato yellow leaf curl virus (TYLCV), one of the most devastating viruses in cultivated tomatoes in the world [4]. *B*. *tabaci* is a complex of 11 well-defined high-level groups consisting of at least 36 putative species identified based on mtCOI (mitochondrial cytochrome oxidase I) [5, 6]. These putative species are morphologically indistinguishable and differ in host range, virus transmission, fecundity, and insecticide resistance [7, 8]. Two major global putative species of *B*. *tabaci*, MEAM1 (Middle East-Asia Minor 1, formerly known as biotype B or *B*. *argentifolii*) and MED (Mediterranean, formerly known as biotype Q), are highly invasive and colonize large areas worldwide [9]. Three putative species (MEAM1, MED, and JpL (*Lonicera japonica*)) of the *B*. *tabaci* species complex are present in Korea. MEAM1 and MED were first detected in 1998 and 2004 [10, 11], respectively. JpL was first recorded in 2014 [12]. Currently, MED is predominant in most regions of the country, and MEAM1 and JpL are found only in a restricted region [12, 13].

Understanding the population genetic structure of a pest species is important for establishing pest management strategies [14]. Pest population structure assessments are helpful to reveal the origins and spread patterns of a target species [15], to delineate potential boundaries for their control [16], and to provide the statistical ability to differentiate between genetic groups [17], as well as to check whether they have mixed with other populations or not. When all population genetics information based on microsatellite markers is combined with environmental approaches, the construction of a powerful framework for managing *B*. *tabaci* is facilitated [18].

Over the past decades, various molecular genetics tools have considerably extended the boundary of population genetics [19]. Diverse DNA markers for insect genetics research (i.e., the amplified fragment length polymorphism (AFLP) marker, expressed sequence tags (EST), mitochondrial DNA (mtDNA), microsatellites, and random amplified polymorphic DNA (RAPD) [20]) have been identified and developed to determine the population genetic structure of a species. Among them, microsatellites are especially popular genetic markers because of their co-dominance, high abundant variation and polymorphism rates, multiple alleles, and quick allele detection by a wide variety of methods [21]. Microsatellite markers are also very effective tools in population genetic studies for insect species [22, 23]. Through molecular genetic diagnosis using population genetic analyses, effective control can be achieved in a short time at a low cost [24]. Different microsatellite markers were employed in several recent studies [25–29] to investigate the population genetic structure, genetic differentiation, genetic evolution, gene flow, and dispersal pattern of *B*. *tabaci* over relatively large geographic scales.

In this study, the population genetic structures and diversities of *B*. *tabaci* MED from tomato greenhouses were identified and their genetic relationships in Korea were examined.

## Materials & methods

### *B*. *tabaci* sampling

**Fig 1:**
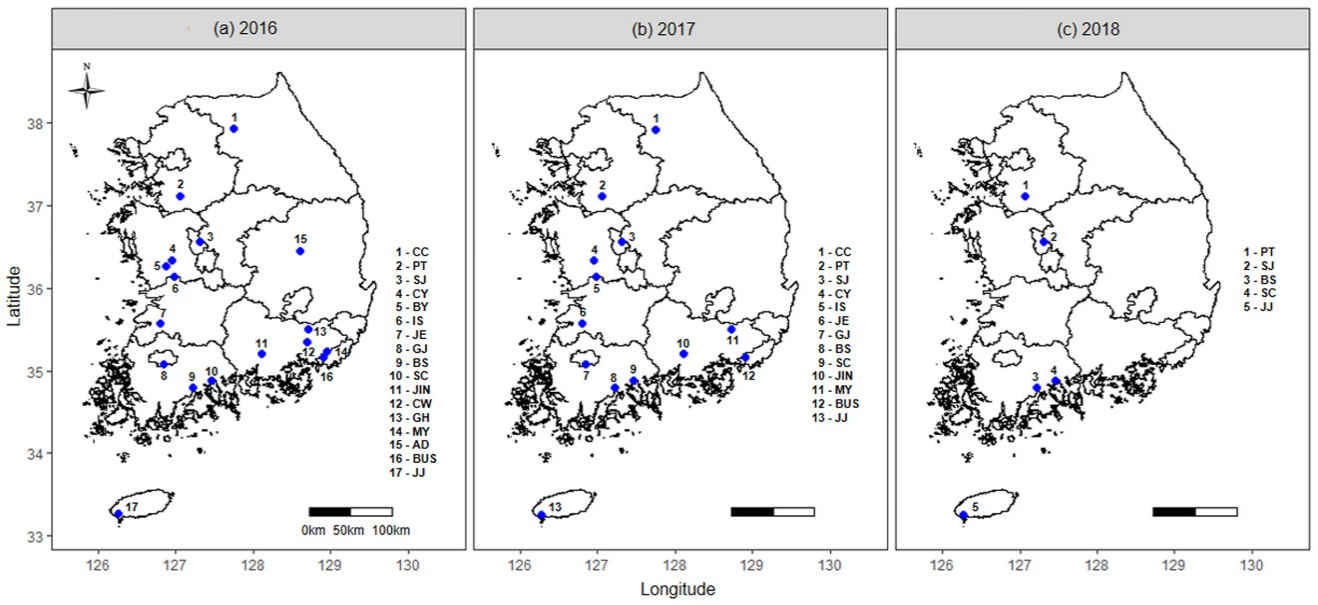
*B*. *tabaci* MED sampling sites (see Table 1 for details) in Korea from (a) 2016, (b) 2017, and (c) 2018.

In total, 1,145 *B*. *tabaci* female adults were collected from 35 commercial tomato greenhouses in Korea using an aspirator during 2016–2018 (17 populations in 2016, 13 populations in 2017, and five populations in 2018) (Fig 1 and Table 1). The *B*. *tabaci* samples were collected from tomatoes plants at least 1 m apart to avoid the collection of full siblings in the greenhouses. All individual samples were preserved in 99.8% ethanol before DNA extraction.

**Table 1:** Details of sampling information of *B*. *tabaci* MED in Korea.

Sample site	Population	Collection date	GPS coordinates	Host plant	Sample size	GenBank accession No.
Seogwipo-si	16’JJ	2016-04-25	37°27′39.0″N,126°57′28.0″E	Tomato	40	HM802268
	17’JJ	2017-04-19	33°15′15.0″N,126°16′09.0″E		20	KY249477
	18’JJ	2018-10-10	33°15′15.0″N,126°16′09.0″E		20	KY249414
Jinju-si *	16’JIN	2016-05-25	35°12′40.0″N,128°06′56.0″E	Tomato	40	EU386987
	17’JIN	2017-06-07			30	EF694108
Changwon-si	16’CW	2016-05-25	35°20′37.0″N,128°42′04.0″E	Tomato	40	KY468417
Busan *	16’BUS	2016-05-25	35°10′18.0″N,128°54′56.0″E	Tomato	40	FJ375358
	17’BUS	2017-06-09			30	HM597869
Gimhae-si	16’GH	2016-05-26	35°14′06.0″N,128°57′42.0″E	Tomato	40	EU263626
Miryang-si *	16’MY	2016-05-26	35°30′08.0″N,128°43′18.0″E	Tomato	40	EU760729
	17’MY	2017-06-08			30	HM597849
Jeongeup-si *	16’JE	2016-06-01	35°34′28.0″N,126°48′07.0″E	Tomato	40	EF667474
	17’JE	2017-06-20			30	EU263630
	18’JE	2018-07-11			20	KY249401
Suncheon-si *	16’SC	2016-06-01	37°27′39.0″N,126°57′28.0″E	Tomato	40	MH357338
	17’SC	2017-06-19			30	KY468420
	18’SC	2018-07-11			20	HM597847
Gwangju *	16’GJ	2016-06-02	35°04′31.0″N,126°51′11.0″E	Tomato	40	KY468410
	17’GJ	2017-06-20			30	KY468415
Boseong-gun *	16’BS	2016-06-02	34°47′33.0″N,127°13′15.0″E	Tomato	40	EU263629
	17’BS	2017-06-19			30	HM597859
Iksan-si	16’IS	2016-06-09	36°08'21.0"N,126°58'59.0"E	Tomato	40	HM597859
	17’IS	2017-06-20	36°08′20.0″N,126°58′55.0″E		15	EU427722
Andong-si	16’AD	2016-06-09	36°27′23.0″N,128°36′11.0″E	Tomato	40	KP137475
Buyeo-gun	16’BY	2016-06-30	36°15′60.0″N,126°52′49.0″E	Tomato	40	MH357340
Cheongyang-gun *	16’CY	2016-06-30	36°20′21.0″N,126°57′18.0″E	Tomato	40	EU760736
	17’CY	2017-06-21			30	KY249451
Sejong-si	16’SJ	2016-06-30	36°34′11.6″N,127°19′02.8″E	Tomato	40	KY249434
	17’SJ	2017-06-19	36°34′19.0″N,127°18′40.0″E		30	MG565975
	18’SJ	2018-07-12	36°34′19.0″N,127°18′40.0″E		20	EU376987
Chuncheon-si	16’CC	2016-07-29	37°56′02.9″N 127°44′57.7″E	Tomato	40	MH357339
	17’CC	2017-06-29	37°55′38.0″N 127°45′15.0″E		30	KY468408
Pyeongtaek-si	16’PT	2016-08-05	37°07′20.0″N,127°03′29.0″E	Tomato	40	MH357340
	17’PT	2017-06-26	37°07′25.0″N,127°03′14.0″E		30	MH205752
	18’PT	2018-08-10	37°07′20.0″N,127°03′29.0″E		20	KY249438

*Same tomato greenhouse during two or three years

### Molecular methods

#### DNA extraction

Genomic DNA (gDNA) extraction was performed using a Qiagen Gentra Puregene Tissue Kit (Qiagen, Gaithersburg, MD, USA) according to the manufacturer’s instructions. Since *B*. *tabaci* is a haplo-diploid species, producing male progeny from unfertilized eggs and female progeny from fertilized eggs [30], only adult females were used for the genetic analysis of each individuals. The extracted gDNA samples were finally stored at -20 °C until use. DNA quantification was performed with ND-1000 spectrophotometer (Nanodrop Technologies, Wilmington, DE, USA).

#### Identification of *B*. *tabaci* putative species

Two individuals per population were randomly selected in order to identify the *B*. *tabaci* putative species. A fragment of the mtCOI gene was PCR-amplified using the primer pair C1-J-2195 (5’-TTGATTTTTTGGTCATCCAGAAGT-3’) and L2-N-3014 (5’-TCCAATGCACTAATCTGCCATATTA-3’) [31]. All PCR reactions were conducted using 1 μl forward primer (10 pmol/μl), 1 μl reverse primer (10 pmol/μl), and 2 μl template DNA in 20 μl reaction volumes consisting of 25 mM dNTPs, 10 mM Tris-HCl (pH 9), 30 mM KCl, 1.5 mM MgCl_2_, and 1 unit of Taq DNA polymerase using Accupower PCR PreMix (Bioneer, Seoul, Korea). The reaction conditions included an initial denaturation for 5 min at 94 °C, followed by 34 cycles of 1 min each at 94 °C, 1 min at 52 °C, and 1 min at 72 °C, with a final extension for 5 min at 72 °C [12]. PCR products were sent for sequencing to NICEM (Seoul, Korea). Putative species identification was based on direct sequence comparisons using NCBI BLAST (http://blast.ncbi.nlm.nih.gov/Blast.cgi).

#### PCR amplification of eight microsatellites

PCR primers were used to amplify microsatellite DNA loci 11, 53 [32], 68, 145, 177 [22], BT4, BT159 [33], and Bem23 [34] using the individual gDNA of *B*. *tabaci* MED as templates. PCR amplifications for the microsatellite primers and PCR reactions were performed as previously described [22]. A total of 1,145 individuals were genotyped using eight microsatellite loci distributed in two PCR multiplex sets. Two multiplex PCRs were performed for each individual at 10 pmol/μl (multiplex 1 loci: 11, 145, 177, BT4, and BT159; multiplex 2 loci: 53, 68, and Bem23). In order to analyze the length of the PCR products using a laser detection system, some of the forward and reverse primers were labeled with a fluorescent dye. The rTaq PCR kit (Takara Bio Inc., Kyoto, Japan) was used for these reactions. The total reaction volume was 10 μl, which contained 2.9 μl or 4.1 μl (multiplex 1: 2.9 μl, multiplex 2: 4.1 μl) distilled water, 1.0 μl 10X PCR buffer, 1.0 μl 2.5mM dNTP mixture, 0.2 μl of each primer, 0.1 μl of Taq polymerase, and 2.0 μl template DNA. The multiplex PCR products were analyzed using an ABI 3730xl (Applied Biosystems Inc., Foster, CA, USA). Allele size was detected using GENEMAPPER v.3.7 (Applied Biosystems Inc.). Multiplex 1 was amplified in PTC100 Thermocyclers (MJ Research, Waltham, MA, USA) as follows: 15 min at 94 °C, followed by 40 cycles for 30 s at 94 °C, 1 min 30 s at 57 °C, 1 min at 72 °C, ending with 30 min at 60 °C. Multiplex 2 was amplified as above except that the annealing temperature was increased from 57 to 60 °C. PCR was carried out as described by Dalmon et al. [22]. The 1 μl PCR product was diluted with 8.5 μl of Hi-Di formamide (Applied Biosystems Inc.) and 0.5 μl Genescan ROX-500 size standard (Applied Biosystems Inc.).

### Analyses of genetic diversity

GENEPOP v.4.0 [35] and Micro-Checker v.2.2.3 [36, 37] were used to determine the microsatellite data for scoring errors, allelic dropouts, and null alleles. The estimated frequency of null alleles per loci for each population was calculated in FreeNa [38] using the expectation maximization (EM) algorithm [39]. Each of the 1,145 collected samples were used to test deviations from Hardy-Weinberg equilibrium (HWE) conditions, the number of alleles (*N*_A_), allele size range, and the observed (*H*_O_) and expected heterozygosities (*H*_E_), and the inbreeding coefficient (*F*_IS_) were computed using GenAlEx v.6.5 [40] and Microsatellite Toolkit [41].

#### Analysis of molecular variance (AMOVA)

AMOVA was performed using GenAlEx v.6.5. AMOVA was used to characterize genetic variation patterns and to estimate variance components. A two-part AMOVA analysis was conducted to check genetic divergence (*F*_ST_) as a factor of variation among and within the populations. AMOVA computations were performed with 999 permutations to test for significance.

### Analyses of genetic structure

The number of genetic clusters (*K*) was estimated in STRUCTURE v.2.3.2 with 60,000 Markov Chain Monte Carlo (MCMC) steps and a burn-in period of 600,000. The log-likelihood estimate was run for *K* = ranges from 1 to 10 with ten replicates each. They were used to determine the number of clusters based on a combination of the mean estimated Ln probability of the data [42] and the second-order rate of change in the log-probability of the data (*ΔK*) [43]. The Evanno method was then implemented in STRUCTURE HARVESTER Web v.0.6.93 [44].

### Principal coordinate analysis (PCoA)

PCoA was conducted between multi-locus genotypes in all individuals. The codominant-genotypic option of GeneAlex v.6.5 was used to calculate the similarity genetic distance matrix [40]. The PCoA plot was based on factor scores along the two principal axes (axis 1 and 2) and enabled the visualization of population differences.

#### Discriminant analysis of principal components (DAPC)

DAPC was performed in the ‘*adegenet*’ package [45] of R software v.3.5.1 (R Development Core Team, 2018) to identify an optimal number of genetic clusters to describe the data. DAPC is a multivariate algorithm, similar to principal component analysis (PCA) that identifies genetic clusters and can be used as an efficient genetic clustering tool [46]. The number of clusters was identified based on Bayesian information criterion (BIC). If the value of BIC is positive and low, it is a suitable model. When the BIC value is negative, a high number is a suitable model.

#### Isolation by distance (IBD)

The Mantel test [47] was performed to assess isolation by distance. The relationship between pairwise geographic distance (Ln km) and pairwise genetic distance in terms of *F*_ST_/(1-*F*_ST_) with 1,000 random permutations was conducted using the GenAlEx v.6.5, GENEPOP v.4.0, and ‘*ade4’* package [48] of R software v.3.5.1. The IBD graph was generated by using the R software v.3.5.1 with ‘*ggpolt2*’ package.

#### Bottleneck test

The BOTTLENECK v.1.2.02 [49] was used to detect the effect of a recent reduction in all population sizes. The possibility of bottleneck events in the 35 populations was examined using a one-tailed Wilcoxon signed-rank test under three mutation models, the infinite allele model (IAM), the two-phase model (TPM), and the stepwise mutation model (SMM) (parameters for TPM: variance = 30.0%, probability = 70.0%, 1,000 replications). The Wilcoxon signed-rank test has been shown to be effective and reliable when eight microsatellite loci are analyzed [49].

#### Pairwise comparisons of fixation index (*F*_ST_)

To assess the level of genetic differentiation between the samples, pair-wise fixation index (*F*_ST_) value estimates were computed using GENEPOP v.4.0. To correct for null alleles, pairwise estimators of *F*_ST_ values were calculated from each microsatellite dataset that potentially harbored null alleles using the excluding null alleles (ENA) correction method (*F*_ST-ENA_) following 1,000 bootstrapping permutations over the loci. The ENA correction method was used to obtain unbiased pairwise *F*_ST_ values using FreeNA. To investigate the relationship between the genetic distance revealed by the *F*_ST_ values and geographic distance, an isolation-by-distance analysis was performed using a regression of *F*_ST_/(1-*F*_ST_) values against the logarithm of the geographical distance (km) between the populations. Significance of the correlation between the two data matrices was assessed using a Mantel test with 1,000 permutations. This was performed with the ISOLDE program implemented in GENEPOP v.4.0.

## Results

### Identification of the *B*. *tabaci* populations

All *B*. *tabaci* individuals collected were successfully sequenced and analyzed. Approximately 810 bp of the mtCOI gene was amplified from *B*. *tabaci* individuals by PCR. All populations identified belonged to the MED (Q1) species based on representative samples.

### Genetic diversity

The values of the genetic diversity indexes for the Korea populations of *B*. *tabaci* MED are shown in Table 2. There were one to eight alleles per loci in the eight microsatellites and the estimated average frequency of null alleles ranged from 0.031 to 0.407 among the 35 populations. The average number of alleles per population (*N*_A_) ranged from 2.000 (17’JIN) to 5.875 (16’SJ). The expected heterozygosity (*H*_E_) ranged from 0.218 (16’JJ) to 0.600 (16’PT), whereas the observed heterozygosity (*H*_O_,) ranged from 0.061 (16’CW) to 0.580 (16’IS). The value of *H*_E_ in each population was higher than the value of *H*_O_, except for 12 populations that showed negative values for *F*_IS_. The estimator of the fixation index inbreeding coefficient (*F*_IS_) ranged from -0.391 (17’CC) to 0.872 (16’CW). A positive value for *F*_IS_ indicates the presence of heterozygotic deficiencies, whereas a negative value indicates the presence of homozygotic deficiencies. The analysis of genetic diversity for all different eight microsatellite loci of *B*. *tabaci* MED screened is given in S1 Table.

**Table 2:** Genetic diversity of the *B*. *tabaci* MED populations.

Population	N	*N*_A_	*H*_E_	*H*_O_	*F*_IS_	*F*_null_
16'JJ	40	2.625	0.218	0.160	0.266	0.241
16'JIN	40	5.500	0.423	0.274	0.353	0.217
16'CW	40	3.500	0.480	0.061	0.872	0.393
16'BUS	40	2.625	0.407	0.118	0.710	0.407
16'GH	40	3.250	0.414	0.159	0.614	0.327
16'MY	40	4.625	0.459	0.107	0.768	0.307
16'JE	40	4.250	0.478	0.337	0.295	0.296
16'SC	40	4.625	0.458	0.282	0.383	0.184
16'GJ	40	2.875	0.462	0.231	0.499	0.284
16'BS	40	4.750	0.521	0.187	0.642	0.292
16'IS	40	5.750	0.549	0.580	-0.057**	0.174
16'AD	40	5.125	0.486	0.272	0.440	0.284
16'BY	40	3.000	0.256	0.136	0.466	0.231
16'CY	40	2.875	0.391	0.180	0.540	0.031
16'SJ	40	5.875	0.594	0.148	0.751	0.333
16'CC	40	5.625	0.445	0.237	0.468	0.255
16'PT	40	5.500	0.600	0.264	0.560	0.300
17'JJ	20	3.000	0.369	0.391	-0.058**	0.268
17'JIN	30	2.000	0.246	0.209	0.150	0.238
17'MY	30	3.375	0.378	0.388	-0.026**	0.331
17'BUS	30	3.250	0.406	0.304	0.251	0.329
17'SJ	30	3.750	0.409	0.417	-0.020**	0.211
17'SC	30	2.875	0.376	0.373	0.010*	0.323
17'BS	30	3.375	0.325	0.339	-0.041**	0.259
17'GJ	30	3.000	0.443	0.425	0.041*	0.382
17'JE	30	3.375	0.394	0.499	-0.265**	0.308
17'IS	15	3.000	0.379	0.426	-0.123**	0.272
17'CY	30	3.000	0.356	0.299	0.160	0.406
17'PT	30	3.250	0.424	0.513	-0.211**	0.240
17'CC	30	2.625	0.387	0.539	-0.391**	0.343
18'SC	20	2.500	0.368	0.413	-0.122**	0.303
18'BS	20	2.875	0.302	0.319	-0.054**	0.273
18'SJ	20	3.375	0.420	0.413	0.019*	0.175
18'PT	20	4.000	0.546	0.250	0.542	0.304
18'JJ	20	2.375	0.286	0.350	-0.225**	0.292

N, number of individuals sampled; *N*_A_, Mean number of alleles per population; *H*_E_, Mean expected heterozygosity; *H*_O_, Mean observed heterozygosity; *F*_IS_, Mean fixation index inbreeding coefficient; and *F*_null_, average proportion of Homozygous for null allele. Significance *F*_IS_ value is obtained after 1,000 permutation tests (*p < 0.05; **p < 0.01).

#### AMOVA

AMOVA among the 35 *B*. *tabaci* MED populations showed that 48.0% of the total genetic variation was accounted for by variation among the populations and 52.0% of the variation was accounted for by individual variation within the populations (Table 3). The AMOVA results revealed a relatively high proportion of variation among the populations.

**Table 3:** Analysis of molecular variance (AMOVA) for the 35 *B*. *tabaci* MED populations collected from different regions in Korea using eight microsatellite markers.

Source of variation	Degrees of freedom	Sums of squares	Mean sums of squares	Estimated variance	% of variation	*p*-value
Among population	34	5557.909	163.468	4.845	48.0%	0.01
Within population	1110	5820.817	5.244	5.244	52.0%	
Total	1144	11378.726		10.089	100%	

Significant at *p* < 0.01 (based on 999 permutations)

#### Genetic relationships and population structure analysis

**Fig 2:**
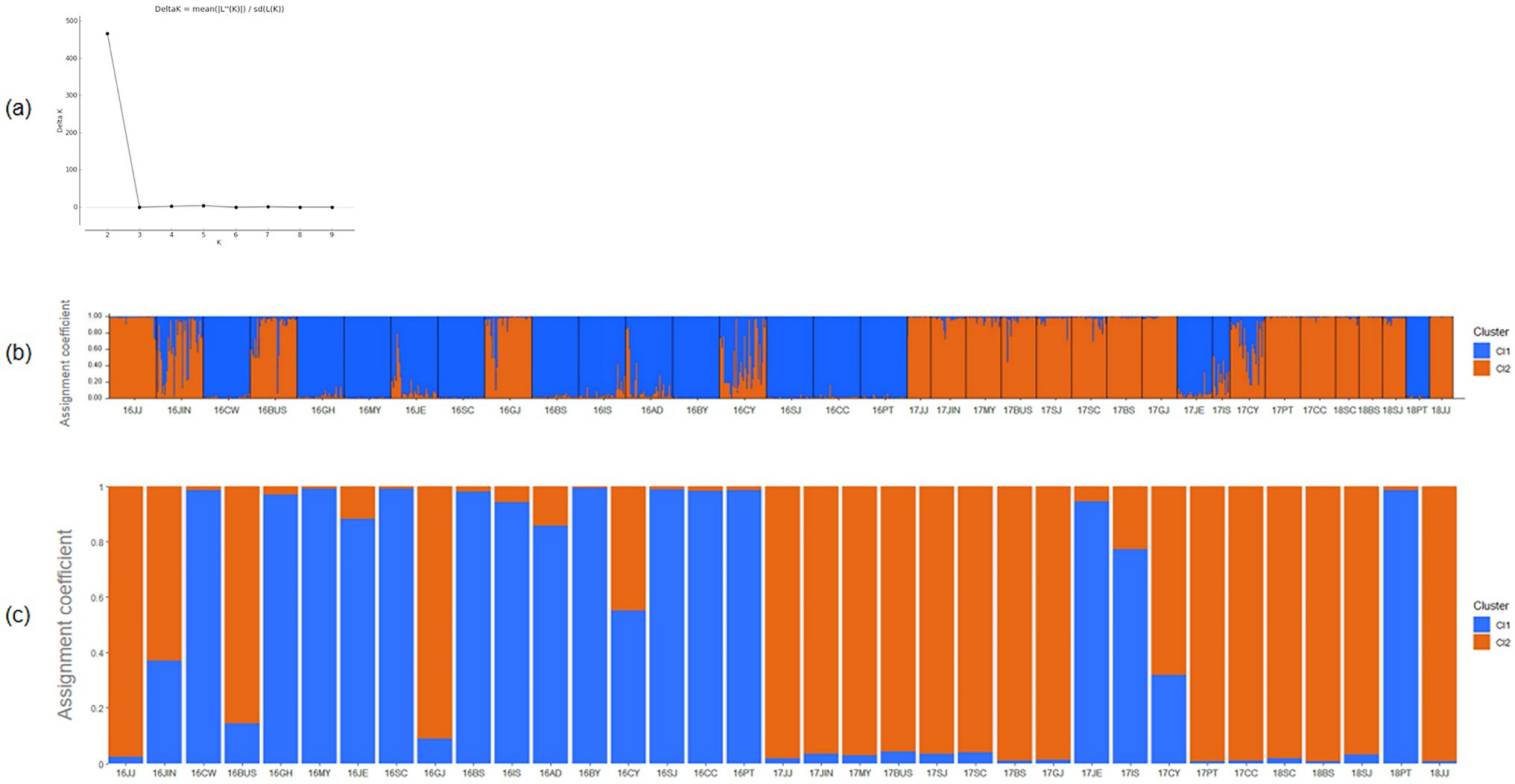
Scatter plots of Δ*K* = 2. (a) The maximum value among the genotypes was 466.35 at Δ*K* = 2, using Δ*K* = m(|L“K|) / s[L(K)]. Bar plot of the population structure for *B*. *tabaci* from 35 populations in Korea (b) using STRUCTURE v.2.3.2 and (c) R software v.3.5.1. Each population is represented by a vertical line with different colors representing the probabilities assigned to each of the genetic clusters.

**Fig 3:**
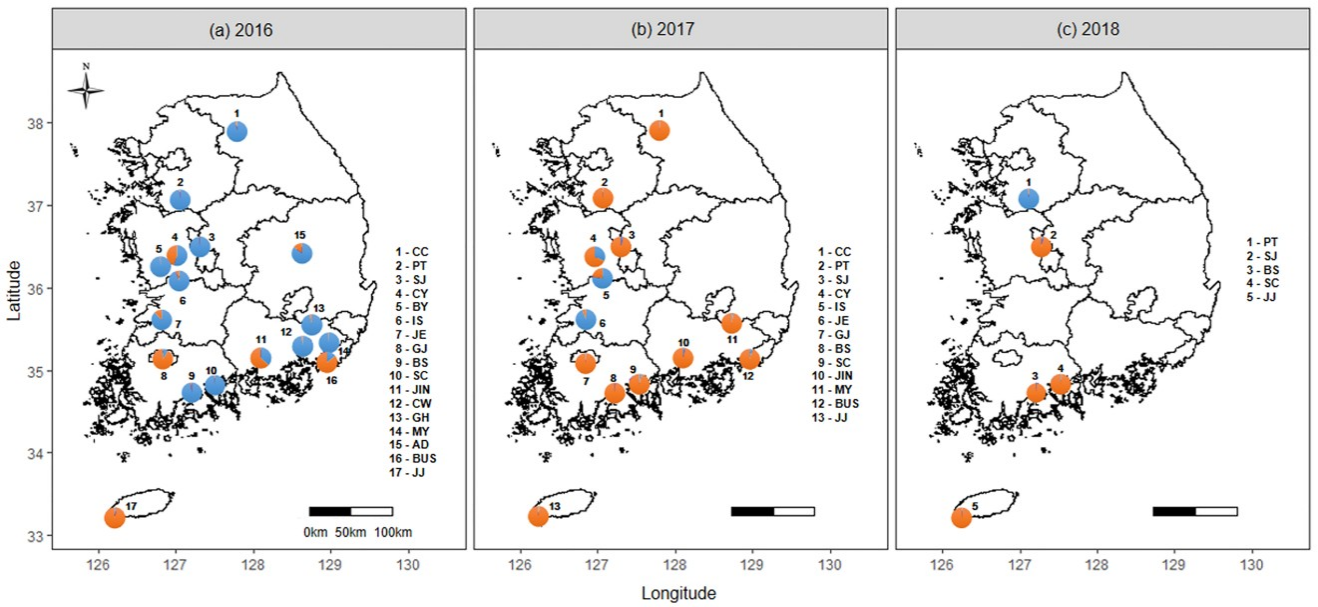
Bayesian clustering results from the structure for all samples (*K* = 2). The geographical distribution of the population and the genetic structure of the *B*. *tabaci* MED in Korea revealed by STRUCTURE analysis in samples from (a) 2016, (b) 2017, and (c) 2018. Genetic changes were observed in six of the populations from 2016 to 2017. The maps were created by using the R software v.3.5.1.

The genetic structure analysis of 35 *B*. *tabaci* MED populations using eight microsatellite marker genotypes revealed two dominant genetic clusters. The highest likelihood value was obtained for *K* = 2 (Fig 2a). The 16 populations (16’CC, 16’PT, 16’SJ, 16’BY, 16’CY, 16’IS, 16’JE, 16’BS, 16’SC, 16’CW, 16’GH, 16’MY, 16’AD, 17’IS, 17’JE, 18’PT) formed one cluster, and 19 populations (16’JIN, 16’GJ, 16’BUS, 16’JJ, 17’CC, 17’PT, 17’SJ, 17’CY, 17’GJ, 17’BS, 17’SC, 17’JIN, 17’MY, 17’BUS, 17’JJ, 18’SJ, 18’JE, 18’BS, 18’SC) formed the other cluster (Fig 2b and 2c). The populations of *B*. *tabaci* MED converged rapidly into one cluster (orange color) over time (Fig 3).

#### PCoA of *B*. *tabaci* MED

**Fig 4:**
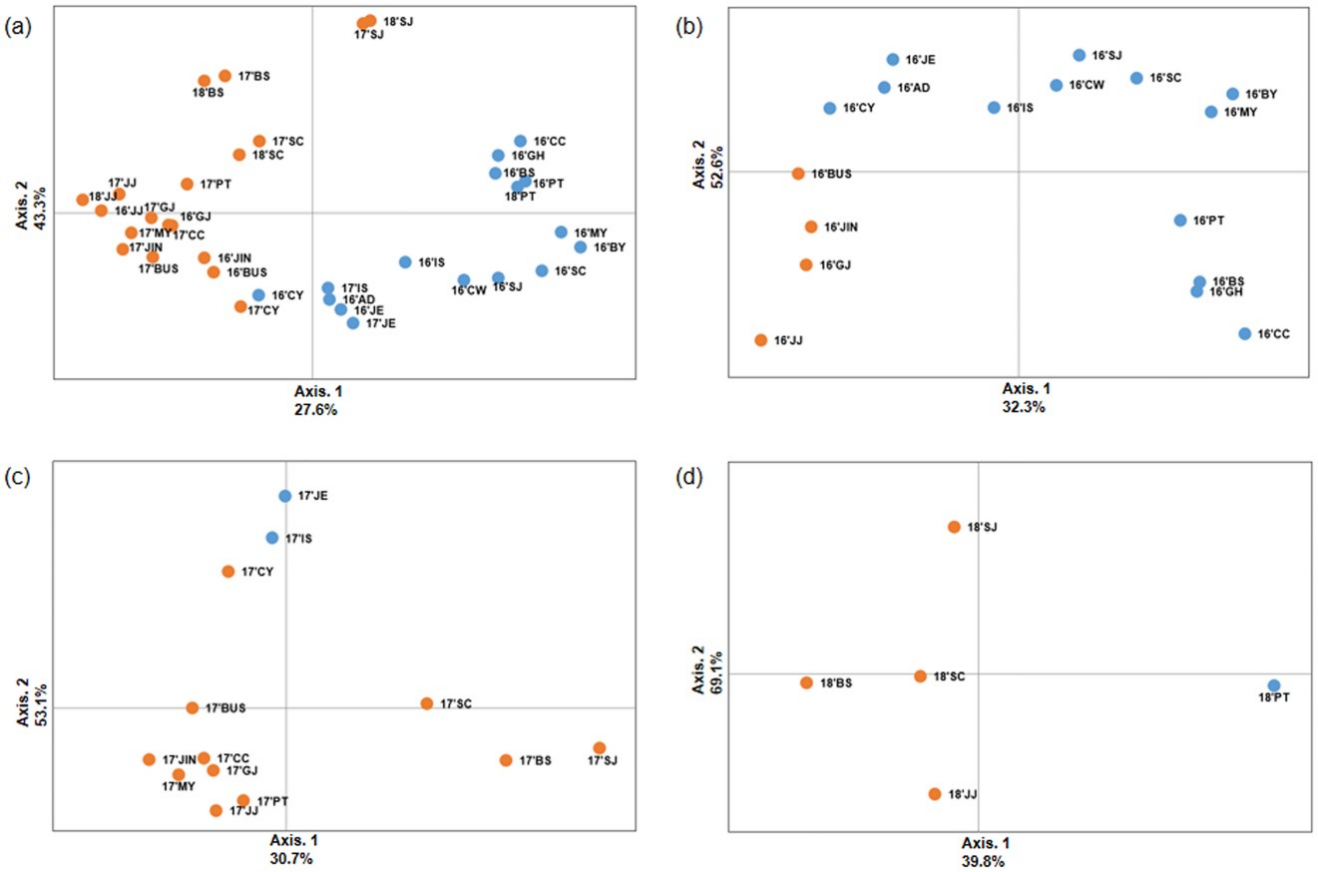
Principal component analysis (PCoA) plotting the relationships of 35 *B*. *tabaci* MED population samples. Each axis indicates the percentage of the total variations. STRUCTURE with marked color is the same as one of Bayesian clustering (blue and orange colors) from (a) 2016–2018, (b) 2016, (c) 2017, and (d) 2018.

Principal component analysis of the 35 *B*. *tabaci* MED populations showed that the first principal components accounted for 27.6% of the total variation, followed by the second component, which accounted for 43.3% of the variation (Fig 4a). The first and second components of PCoA for each year are as follows: 32.3%, 52.6% for 2016 (Fig 4b), 30.7%, 53.1% for 2017 (Fig 4c), and 39.8%, 69.1% for 2018 (Fig 4d), respectively.

#### DAPC

**Fig 5:**
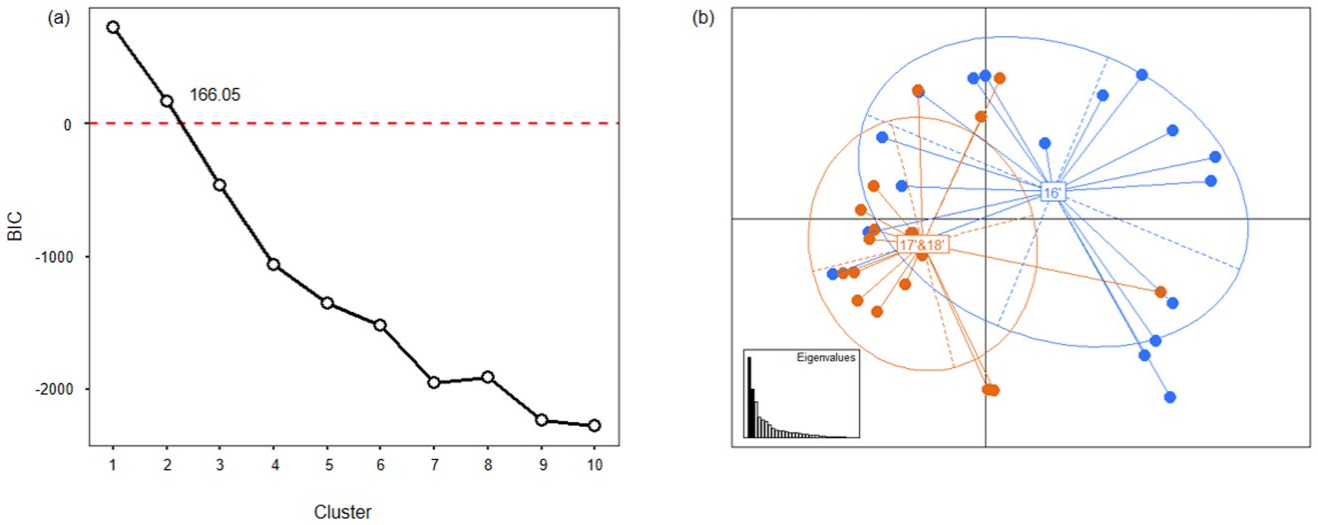
Discriminant analysis of principal components (DAPC) analysis of 35 *B*. *tabaci* MED populations in Korea. (a) The Bayesian information criteria (BIC) supported two distinct genetic clusters. (b) The eigenvalues of the analysis suggest that the first two components explained the maximum genetic structure of the dataset. Scatter-plot of the distribution of *B*. *tabaci* MED formed two genetic clusters (blue and orange colors).

In DAPC, the elbow in the curve of BIC was at *K* = 2 using the *find*. *cluster* function of R software v.3.5.1 [50]. In this study, the value of BIC was found to be 166.05, which was positive and the smallest value (Fig 5a). The DAPC results showed that the populations of *B*. *tabaci* MED were split into two well-differentiated genetic clusters with low overlap between them. The first cluster contained populations from 2016 and the second cluster contained populations from 2017 and 2018 (Fig 5b). The DAPC results agreed with the STRUCTURE results.

#### IBD

**Fig 6:**
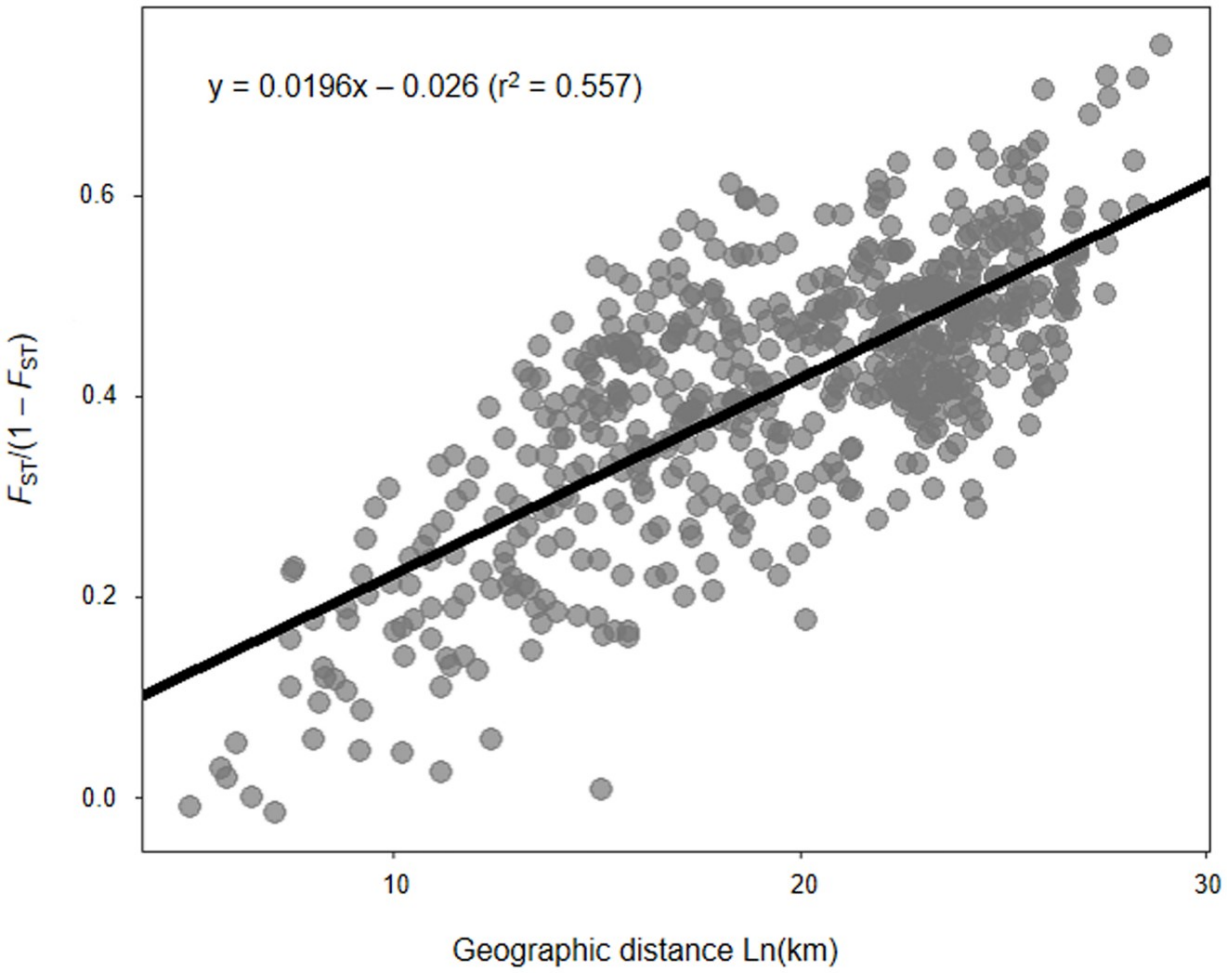
Relationship between genetic distance and the log of the geographical distance for *B*. *tabaci* MED. The line represents the regression line and circles represent the logarithm transformation of distance (*p* = 0.01, 1,000 permutations).

A significant correlation was detected between genetic and geographic distances in the *B*. *tabaci* MED populations based on the Mantel tests of IBD (*r*^2^ = 0.557; *p* = 0.01), indicating a pattern of isolation by distance (Fig 6). Multiple points in the scatterplot fit to the linear regression along the geographic distance range. This result indicates that gene flow between population increases with geographic distance. IBD analysis revealed that geographic distance had an effect on the population structure of the *B*. *tabaci*.

#### Bottleneck test

The mode-shift analysis of bottleneck test, a signature of recent population reduction was found only for the 16’GJ and 18’PT populations (Table 4). Departure from mutation-drift equilibrium was observed in two populations, indicating that they remained relatively unstable in recent evolutionary history. Significant heterozygosity excess (Wilcoxon test *p*-values) was detected in eight populations under the IAM (16’CW, 16’BUS, 16’GJ, 16’CY, 17’BUS, 17’GJ, 17’CY, 18’PT) and two populations under the TPM (16’GJ, 17GJ), which accounted for 22.8% and 5.7% of the Korea populations (Table 4 bolded numbers), respectively. Under the SMM, however, significant heterozygosity excess was not detected in any population.

**Table 4:** Wilcoxon signed-rank tests for heterozygosity excess for the 35 *B*. *tabaci* MED populations.

Population	WILCOXON Tests * (Heterozygosity Excess *p*-values)			Mode-Shift
	IAM	TPM	SMM	
16'JJ	0.94531	0.97266	0.98047	Normal
16'JIN	0.84375	0.99609	1.00000	Normal
16'CW	**0.03711**	0.52734	0.72656	Normal
16'BUS	**0.01953**	0.19147	0.52734	Normal
16'GH	0.12500	0.37109	0.96289	Normal
16'MY	0.67969	0.97266	0.99023	Normal
16'JE	0.14844	0.59375	0.07813	Normal
16'SC	0.52734	0.76953	0.99609	Normal
16'GJ	**0.00781**	**0.01172**	0.05469	Shifted mode
16'BS	0.32031	0.97266	0.99414	Normal
16'IS	0.27344	0.76953	0.99023	Normal
16'AD	0.62891	0.99414	1.00000	Normal
16'BY	0.37109	0.97266	1.00000	Normal
16'CY	**0.01953**	0.15625	0.52734	Normal
16'SJ	0.32031	0.80859	0.98633	Normal
16'CC	0.72656	0.97266	0.99609	Normal
16'PT	0.12500	0.37109	0.84375	Normal
17'JJ	0.42188	0.76953	0.84375	Normal
17'JIN	0.28906	0.46875	0.65625	Normal
17'MY	0.46875	0.76563	0.96094	Normal
17'BUS	**0.03906**	0.65625	0.94531	Normal
17'SJ	0.52734	0.67969	0.98047	Normal
17'SC	0.32031	0.52734	0.76953	Normal
17'BS	0.65625	0.96094	0.97266	Normal
17'GJ	**0.00781**	**0.01563**	0.07813	Normal
17'JE	0.40625	0.81250	0.94531	Normal
17'IS	0.40625	0.65625	0.81250	Normal
17'CY	**0.03906**	0.28906	0.94531	Normal
17'PT	0.34375	0.46875	0.46875	Normal
17'CC	0.15625	0.47266	0.76953	Normal
18'SC	0.05469	0.23438	0.28906	Normal
18'BS	0.57813	0.78125	0.96094	Normal
18'SJ	0.23438	0.34375	0.65625	Normal
18'PT	**0.01953**	0.18750	0.40625	Shifted mode
18'JJ	0.34375	0.65625	0.65625	Normal

Infinite allele model (IAM), two-phase model (TPM), and stepwise mutation model (SMM) for detection of a recent population bottleneck event within each *B*. *tabaci* MED population.

*One-tailed Wilcoxon signed-rank test; Bolded numbers indicate they are significant at *p* < 0.05.

#### Pairwise comparisons of fixation index (*F*_ST_)

The fixation index (*F*_ST_) reflects the degree of genetic differentiation among the populations. *F*_ST_ is close to 0 when the genetic variation shows no difference in fixation among the populations. It is close to 1 when genetic differentiation is high. In this study, the *F*_ST_ values ranged from -0.0155 to 0.7501 and the ENA-corrected *F*_ST_ values ranged from -0.0139 to 0.7327 among the populations (Table 5). The highest *F*_ST_ value was detected between the 16’JJ and 16’BY populations (0.7327). The lowest *F*_ST_ value was found between the 17’SJ and 18’SJ populations (-0.0139). In practice, an *F*_ST_ value of 0.00–0.05 indicates low differentiation and *F*_ST_ values > 0.15 indicate a high level of differentiation. Negative *F*_ST_ values are allowed because correlations vary from -1 to +1 [51]. As a result, most *B*. *tabaci* in Korea showed high levels of differentiation.

**Table 5:** Pairwise *F*_ST_ values based on variation at eight microsatellite loci between the *B*. *tabaci* MED populations.

	**16'JJ**	**16'JIN**	**16'CW**	**16'BUS**	**16'GH**	**16'MY**	**16'JE**	**16'SC**	**16'GJ**	**16'BS**	**16'IS**	**16'AD**	**16'BY**	**16'CY**	**16'SJ**	**16'CC**	**16'PT**	**17'JJ**
**16'JJ**		0.2894	0.5640	0.3427	0.5955	0.6336	0.5115	0.6201	0.2432	0.5249	0.5102	0.4856	0.7501	0.4542	0.5407	0.5687	0.5179	0.0949
**16'JIN**	0.2487		0.3983	0.1616	0.4852	0.4822	0.2634	0.4194	0.1808	0.4207	0.3661	0.2193	0.6080	0.2214	0.3667	0.4630	0.3748	0.2120
**16'CW**	0.5356	0.3746		0.3854	0.4038	0.3810	0.3126	0.3222	0.3871	0.3753	0.3226	0.2892	0.4910	0.3956	0.1769	0.4414	0.3712	0.4565
**16'BUS**	0.3232	0.1343	0.3635		0.4928	0.4916	0.2589	0.4466	0.2229	0.4599	0.3217	0.2005	0.5890	0.1597	0.3524	0.5230	0.4210	0.2364
**16'GH**	0.5750	0.4727	0.3767	0.4713		0.4588	0.4982	0.4018	0.4518	0.3322	0.4360	0.4740	0.5783	0.5415	0.4095	0.2679	0.3062	0.5041
**16'MY**	0.5957	0.4491	0.3105	0.4568	0.3863		0.4042	0.3066	0.5012	0.3076	0.4082	0.4239	0.4962	0.4622	0.3077	0.4060	0.3447	0.5371
**16'JE**	0.4903	0.2438	0.3042	0.2360	0.4778	0.3796		0.2899	0.3520	0.4515	0.3373	0.1653	0.5605	0.1794	0.2595	0.5084	0.3638	0.3963
**16'SC**	0.6078	0.4208	0.3201	0.4449	0.3631	0.2871	0.3052		0.4662	0.4038	0.3726	0.3251	0.5146	0.3628	0.2420	0.3949	0.2365	0.5267
**16'GJ**	0.2337	0.1458	0.3484	0.1597	0.4267	0.4396	0.3243	0.4479		0.4079	0.3568	0.2797	0.5975	0.3004	0.3999	0.4662	0.3857	0.1408
**16'BS**	0.5050	0.4093	0.3140	0.4370	0.2907	0.2504	0.4293	0.3864	0.3734		0.3923	0.4359	0.4639	0.4870	0.3374	0.2334	0.2769	0.4252
**16'IS**	0.4959	0.3545	0.2974	0.3289	0.4141	0.3624	0.3324	0.3668	0.3350	0.3629		0.3021	0.4615	0.3630	0.2219	0.4727	0.3570	0.3991
**16'AD**	0.4574	0.1882	0.2726	0.1810	0.4452	0.3766	0.1237	0.3191	0.2588	0.4001	0.2916		0.4904	0.1654	0.2954	0.4993	0.3674	0.3683
**16'BY**	0.7327	0.6041	0.4835	0.6019	0.5491	0.4690	0.5580	0.5133	0.5848	0.4452	0.4461	0.4874		0.5714	0.4263	0.5271	0.4362	0.6802
**16'CY**	0.4280	0.1899	0.3687	0.1489	0.5053	0.4182	0.1626	0.3460	0.2527	0.4528	0.3429	0.1610	0.5633		0.3328	0.5438	0.4008	0.3323
**16'SJ**	0.5188	0.3442	0.1298	0.3333	0.3651	0.2527	0.2440	0.2380	0.3489	0.2789	0.2008	0.2587	0.4266	0.2997		0.4219	0.2885	0.4372
**16'CC**	0.5446	0.4558	0.3950	0.5085	0.2155	0.3435	0.4867	0.3608	0.4410	0.2069	0.4456	0.4658	0.4969	0.5128	0.3771		0.2052	0.4913
**16'PT**	0.5045	0.3676	0.3051	0.3968	0.2395	0.2828	0.3490	0.2172	0.3495	0.2447	0.3328	0.3362	0.4351	0.3743	0.2344	0.1729		0.4150
**17'JJ**	0.1137	0.1934	0.4268	0.2116	0.4815	0.4971	0.3769	0.5198	0.1342	0.4088	0.3835	0.3419	0.6658	0.3078	0.4135	0.4781	0.4049	
**17'JIN**	0.1919	0.1463	0.4985	0.2682	0.5684	0.5526	0.4082	0.5464	0.2399	0.4907	0.4580	0.3705	0.7034	0.2922	0.4741	0.5344	0.4787	0.1724
**17'MY**	0.1117	0.1209	0.4169	0.1620	0.4798	0.4859	0.3333	0.4927	0.1089	0.4120	0.3816	0.3040	0.6444	0.2539	0.3960	0.4731	0.4040	**0.0466**
**17'BUS**	0.2123	0.1147	0.3807	**0.0491**	0.4710	0.4581	0.2590	0.4534	0.1304	0.4159	0.3458	0.2388	0.6129	0.1434	0.3510	0.4811	0.3874	0.1098
**17'SJ**	0.5808	0.4871	0.4695	0.4679	0.4718	0.4656	0.4873	0.4982	0.4380	0.4285	0.4474	0.4720	0.6113	0.4870	0.4215	0.4767	0.3945	0.4584
**17'SC**	0.5147	0.3346	0.4464	0.3637	0.5174	0.4587	0.3982	0.4443	0.3429	0.4546	0.3993	0.3630	0.6230	0.3265	0.3838	0.5090	0.3893	0.3870
**17'BS**	0.5535	0.4179	0.4811	0.4274	0.5289	0.5364	0.4585	0.5515	0.3894	0.5132	0.4804	0.4277	0.6750	0.4395	0.4627	0.5422	0.4618	0.4104
**17'GJ**	0.2570	0.1775	0.3932	0.1874	0.4730	0.4779	0.3466	0.4923	**0.0413**	0.4109	0.3746	0.2914	0.6128	0.2695	0.3881	0.4743	0.3875	0.1429
**17'JE**	0.5528	0.3073	0.3220	0.3023	0.5127	0.4031	**0.0499**	0.3069	0.3693	0.4648	0.3589	0.1696	0.5906	0.2140	0.2874	0.5226	0.3958	0.4405
**17'IS**	0.5543	0.3683	0.3920	0.3368	0.5073	0.4588	0.3790	0.4408	0.3433	0.4486	0.1003	0.3096	0.5353	0.3189	0.2951	0.5181	0.3958	0.4252
**17'CY**	0.4606	0.2164	0.3974	0.1638	0.5385	0.4563	0.1890	0.3979	0.2645	0.4869	0.3560	0.1587	0.5969	**0.0245**	0.3400	0.5521	0.4166	0.3233
**17'PT**	0.3562	0.2511	0.4435	0.3172	0.4814	0.4811	0.4080	0.4963	0.1767	0.4039	0.4019	0.3760	0.6363	0.3570	0.4040	0.4634	0.3905	0.2466
**17'CC**	0.2495	0.1793	0.4270	0.2110	0.4729	0.4679	0.3580	0.4688	0.1806	0.4003	0.3679	0.3349	0.6273	0.2505	0.3824	0.4672	0.3999	0.1486
**18'SC**	0.5227	0.3110	0.4460	0.3442	0.5241	0.4650	0.3734	0.4413	0.3369	0.4622	0.3888	0.3383	0.6383	0.3066	0.3724	0.5184	0.3861	0.3809
**18'BS**	0.5666	0.4143	0.4869	0.4249	0.5462	0.5491	0.4596	0.5573	0.3838	0.5249	0.4759	0.4287	0.6976	0.4369	0.4611	0.5563	0.4671	0.4145
**18'SJ**	0.5997	0.4883	0.4632	0.4744	0.4720	0.4572	0.4824	0.4931	0.4375	0.4195	0.4376	0.4663	0.6166	0.4884	0.4114	0.4694	0.3840	0.4664
**18'PT**	0.5779	0.4134	0.3525	0.4479	0.2800	0.3409	0.3892	0.2336	0.3949	0.3150	0.3621	0.3687	0.5085	0.4221	0.2693	0.2308	**0.0098**	0.4621
**18'JJ**	**0.0441**	0.2128	0.4835	0.2585	0.5312	0.5446	0.4286	0.5592	0.1658	0.4604	0.4280	0.3947	0.7042	0.3508	0.4610	0.5147	0.4497	**0.0181**
																		

	**17'JIN**	**17'MY**	**17'BUS**	**17'SJ**	**17'SC**	**17'BS**	**17'GJ**	**17'JE**	**17'IS**	**17'CY**	**17'PT**	**17'CC**	**18'SC**	**18'BS**	**18'SJ**	**18'PT**	**18'JJ**
**16'JJ**	0.2578	0.1192	0.2356	0.6150	0.5451	0.5986	0.2960	0.5896	0.5962	0.4948	0.3874	0.3084	0.5548	0.6104	0.6324	0.5848	**0.0294**
**16'JIN**	0.1569	0.1375	0.1259	0.5103	0.3496	0.4617	0.2111	0.3315	0.3913	0.2578	0.2599	0.1876	0.3223	0.4546	0.5083	0.4060	0.2415
**16'CW**	0.5095	0.4458	0.4164	0.5157	0.4877	0.5196	0.4245	0.3181	0.4082	0.4004	0.4775	0.4553	0.4810	0.5184	0.5052	0.4088	0.5084
**16'BUS**	0.2492	0.1951	0.0575	0.4963	0.3764	0.4729	0.2371	0.3245	0.3243	0.1835	0.3469	0.2198	0.3456	0.4646	0.4976	0.4529	0.2892
**16'GH**	0.5784	0.5009	0.4969	0.5081	0.5537	0.5572	0.4917	0.5378	0.5366	0.5588	0.4988	0.4980	0.5573	0.5690	0.5042	0.3471	0.5467
**16'MY**	0.5740	0.5212	0.4983	0.5172	0.4973	0.5836	0.5208	0.4280	0.4962	0.4795	0.5148	0.5017	0.5021	0.5896	0.5059	0.3903	0.5791
**16'JE**	0.4148	0.3445	0.2818	0.5044	0.4140	0.4878	0.3754	**0.0460**	0.3915	0.2079	0.4202	0.3661	0.3815	0.4831	0.4956	0.3938	0.4450
**16'SC**	0.5424	0.4941	0.4604	0.5176	0.4588	0.5711	0.5021	0.2988	0.4496	0.3931	0.5011	0.4727	0.4527	0.5724	0.5089	0.2592	0.5659
**16'GJ**	0.2699	0.1313	0.1874	0.4769	0.3933	0.4359	**0.0443**	0.4008	0.3797	0.2958	0.2083	0.2246	0.3845	0.4260	0.4724	0.4118	0.1761
**16'BS**	0.4931	0.4296	0.4379	0.4645	0.4848	0.5463	0.4239	0.4909	0.4754	0.5045	0.4108	0.4171	0.4911	0.5509	0.4519	0.3319	0.4716
**16'IS**	0.4670	0.3922	0.3559	0.4677	0.4132	0.5022	0.3855	0.3707	0.1102	0.3558	0.4128	0.3770	0.3983	0.4954	0.4569	0.3778	0.4458
**16'AD**	0.3857	0.3293	0.2689	0.4945	0.3818	0.4609	0.3139	0.1988	0.3046	0.1459	0.3932	0.3530	0.3525	0.4561	0.4854	0.3877	0.4200
**16'BY**	0.7056	0.6539	0.6181	0.6354	0.6359	0.6983	0.6199	0.6044	0.5386	0.5878	0.6455	0.6364	0.6529	0.7170	0.6386	0.5056	0.7192
**16'CY**	0.3006	0.2823	0.1726	0.5237	0.3575	0.4804	0.3114	0.2333	0.3489	**0.0255**	0.3965	0.2837	0.3307	0.4749	0.5234	0.4378	0.3850
**16'SJ**	0.4821	0.4169	0.3793	0.4592	0.4129	0.4940	0.4191	0.2921	0.3021	0.3452	0.4286	0.4015	0.3907	0.4845	0.4445	0.3060	0.4779
**16'CC**	0.5450	0.4867	0.4964	0.5087	0.5366	0.5689	0.4859	0.5531	0.5510	0.5727	0.4741	0.4871	0.5480	0.5791	0.4997	0.2733	0.5232
**16'PT**	0.4813	0.4142	0.4084	0.4249	0.4122	0.4900	0.4037	0.4164	0.4146	0.4235	0.3970	0.4128	0.4073	0.4887	0.4096	**0.0075**	0.4548
**17'JJ**	0.2023	0.0581	0.1409	0.4861	0.4044	0.4507	0.1688	0.4722	0.4612	0.3579	0.2493	0.1886	0.3991	0.4493	0.4911	0.4589	**0.0199**
**17'JIN**		0.1285	0.1053	0.5953	0.4455	0.5641	0.2622	0.4860	0.5104	0.3576	0.3406	0.1569	0.4490	0.5750	0.6073	0.5363	0.2287
**17'MY**	0.1135		0.0856	0.5171	0.3906	0.4737	0.1658	0.4203	0.4321	0.3025	0.2136	0.1086	0.3784	0.4707	0.5184	0.4519	0.0543
**17'BUS**	0.1301	0.0657		0.4967	0.3591	0.4613	0.1887	0.3568	0.3633	0.2021	0.2783	0.1176	0.3405	0.4542	0.4969	0.4413	0.1766
**17'SJ**	0.5704	0.4831	0.4599		0.3850	0.4446	0.4899	0.5469	0.5373	0.5447	0.4717	0.4862	0.4037	0.4541	**-0.0155**	0.4676	0.5422
**17'SC**	0.4435	0.3727	0.3484	0.3222		0.4272	0.4082	0.4687	0.4540	0.3943	0.3823	0.3277	**-0.0004**	0.4328	0.3748	0.4549	0.4694
**17'BS**	0.5205	0.4253	0.4079	0.4077	0.3754		0.4386	0.5402	0.5797	0.5008	0.5057	0.5104	0.4168	**-0.0090**	0.4445	0.5363	0.5206
**17'GJ**	0.2284	0.1256	0.1320	0.4630	0.3733	0.3953		0.4286	0.4022	0.3191	0.2120	0.2379	0.3997	0.4318	0.4860	0.4331	0.2208
**17'JE**	0.4679	0.3964	0.3250	0.5156	0.4386	0.5007	0.3933		0.4438	0.2747	0.4782	0.4358	0.4469	0.5415	0.5435	0.4555	0.5270
**17'IS**	0.4880	0.4007	0.3426	0.4970	0.4279	0.5361	0.3752	0.4175		0.3405	0.4378	0.3959	0.4502	0.5809	0.5320	0.4469	0.5243
**17'CY**	0.3242	0.2654	0.1569	0.5207	0.3746	0.4596	0.2798	0.2546	0.3391		0.4062	0.3054	0.3754	0.4983	0.5447	0.4608	0.4163
**17'PT**	0.3117	0.1993	0.2509	0.4397	0.3531	0.4709	0.1919	0.4545	0.4074	0.3764		0.1761	0.3923	0.5011	0.4647	0.4248	0.2876
**17'CC**	0.1477	0.0869	0.1141	0.4469	0.3146	0.4659	0.1934	0.4144	0.3672	0.2725	0.1557		0.3303	0.5083	0.4860	0.4469	0.2246
**18'SC**	0.4470	0.3613	0.3346	0.3418	**-0.0044**	0.3607	0.3653	0.4206	0.4263	0.3586	0.3649	0.3164		0.4250	0.3953	0.4470	0.4735
**18'BS**	0.5342	0.4254	0.4045	0.4194	0.3840	**-0.0064**	0.3921	0.5037	0.5384	0.4608	0.4690	0.4648	0.3718		0.4541	0.5368	0.5280
**18'SJ**	0.5843	0.4875	0.4637	**-0.0139**	0.3120	0.4080	0.4627	0.5133	0.4931	0.5239	0.4348	0.4476	0.3333	0.4205		0.4512	0.5512
**18'PT**	0.5455	0.4558	0.4368	0.4415	0.4380	0.5129	0.4331	0.4386	0.4391	0.4657	0.4344	0.4461	0.4349	0.5215	0.4316		0.5093
**18'JJ**	0.1705	**0.0431**	0.1401	0.5144	0.4395	0.4731	0.1804	0.4917	0.4764	0.3713	0.2707	0.1681	0.4407	0.4821	0.5255	0.5145	

Significant values (*p* < 0.05) for pairwise *F*_ST_ are in bold.

## Discussion

This study is the first comprehensive genetic structure analysis of *B*. *tabaci* MED (Q1) populations in Korea using eight microsatellite loci. The Korean populations of tomato *B*. *tabaci* MED appeared to be classified into two genetic clusters based on STRUCTURE and DAPC analyses, and their genetic structure converged rapidly into one genetic cluster. This phenomenon was reported previously by Dinsdale et al. [52] in Australia. They reported that the genetic cluster of *B*. *tabaci* rapidly changed even in a period of just four months. The results of this study and those by Dinsdale et al. [52], suggested that one out of the two *B*. *tabaci* MED genetic clusters in Korea might become the dominant species in the future.

This phenomenon could be caused by different fitness between the two *B*. *tabaci* MED genetic clusters in Korea. Although the two *B*. *tabaci* MED genetic clusters might have been mixed when they were first introduced in new areas, one genetic cluster would become dominant if there is fitness difference between them. Fitness difference between two genetic clusters could result from different susceptibilities to insecticides. The use of various insecticides, such as neonicotinoids, organophosphates, and carbamates, has been the main control method for *B*. *tabaci* MED in Korea. Extensive use of these insecticides has rapidly resulted in high levels of insecticide resistance in *B*. *tabaci* MED populations [53]. The two genetic clusters of *B*. *tabaci* MED might have different potentials for developing resistance to different insecticides. This differentiation was partially supported by changing the frequencies and diversity caused by chemical control [54, 55]. Results of the current study also showed low genotype frequencies and diversities, and limited founder or bottleneck effects.

However, the speed of this genetic cluster change in Korea could differ by areas. For example, the Jeju populations showed one genetic cluster of *B*. *tabaci* MED and this trend was maintained during the past three years. However, in the Pyeongtaek area, the genetic cluster of *B*. *tabaci* MED changed every year. The differences in the speed of genetic cluster change could be caused by human-related factors because *B*. *tabaci* has a low dispersal ability over long distances [56]. In the case of Jeju, the *B*. *tabaci* MED populations should not have been affected by other populations because almost all growers produce tomato seedlings themselves and Jeju is isolated because it is an island. On the other hand, the Pyeongtaek tomato growers have purchased tomato seedlings from different nurseries every year. Moreover, the city of Pyeongtaek has one of the most active agricultural trades of all Korean cities. Whitefly populations are generally affected by human activities, such as the movement of infested plants from nurseries, material shipments, and commercial trading, rather than by active flight [54, 57]. Thus, the populations in areas with high human activities and diverse nursery routes (i.e., the Pyeongtaek populations) might show accelerated genetic cluster changes compared to populations in isolated areas with limited nursery routes (i.e., the Jeju populations).

The information on the genetic characteristics of *B*. *tabaci* in areas where it usually occurs should be useful for efficient management of *B*. *tabaci* [58–60]. The genetic structure information gathered from the long-term and large-scale field analysis in this study facilitates a better understanding of the population dynamics of *B*. *tabaci* MED as an invasive pest in Korea. Thus, the results of this study could be a valuable foundation to develop efficient management strategies for *B*. *tabaci* MED in Korea. However, further studies are needed to clearly find the fitness differences between the two *B*. *tabaci* MED genetic clusters in Korea.

## Supporting information

S1 Table Genetic diversity for all different eight microsatellite loci screened for *B*. *tabaci* MED in Korea. ^a^Number of alleles. ^b^Expected heterozygosity. ^c^Observed heterozygosity. ^d^Mean fixation index inbreeding coefficient. ^e^Average proportion of homozygous for null allele.
(XLSX)
